# Efficacy and safety of switching from nevirapine immediate-release twice daily to nevirapine extended-release once daily in virologically suppressed HIV-infected patients: a retrospective cohort study in Taiwan

**DOI:** 10.1186/s12879-017-2371-3

**Published:** 2017-04-11

**Authors:** Chun-Yuan Lee, Hui-Min Chang, Calvin M Kunin, Susan Shin-Jung Lee, Yao-Shen Chen, Hung-Chin Tsai

**Affiliations:** 1 Division of Infectious Diseases, Department of Internal Medicine, Kaohsiung Medical University, Kaohsiung Medical University Hospital, Kaohsiung, Taiwan; 2 Center for Infectious Disease and Cancer Research (CICAR), Kaohsiung Medical University, Kaohsiung, Taiwan; 3 Graduate Institute of Medicine, Kaohsiung Medical University, Kaohsiung, Taiwan; 4 Department of Pharmacy, Kaohsiung Veterans General Hospital, Kaohsiung, Taiwan; 5 Department of Internal Medicine, Ohio State University, Columbus, OH USA; 6 Department of Internal Medicine, University of Arizona, Tucson, AZ USA; 7 Division of Infectious Diseases, Department of Medicine, Kaohsiung Veterans General Hospital, Kaohsiung, Taiwan; 8 Faculty of Medicine, School of Medicine, National Yang-Ming University, Taipei, Taiwan; 9 Graduate Institute of Science Education and Environmental Education, National Kaohsiung Normal University, Kaohsiung, Taiwan

**Keywords:** Antiretroviral therapy, Human immunodeficiency virus, Nevirapine

## Abstract

**Background:**

Whether the non-inferior efficacy and safety results of switching virologically suppressed HIV-1-infected patients from nevirapine immediate-release (NVP-IR) to NVP extended-release (NVP-XR) demonstrated in the TRANxITION study conducted in Europe and North America are also applicable to virologically suppressed HIV-infected Taiwanese patients remains unknown. We evaluated the comparative safety and efficacy of continuing NVP-IR versus switching to NVP-XR in virologically suppressed HIV-infected Taiwanese adults receiving combined antiretroviral therapy (cART) regimens.

**Methods:**

We conducted a retrospective cohort study at Kaohsiung Veterans General Hospital from April 1, 2013, to March 31, 2015. Eighty-four virologically suppressed HIV-infected adults receiving NVP-IR cART were split into two groups: those continuing with NVP-IR (*n* = 49) and those being switched to NVP-XR (*n* = 35). Demographic characteristics, clinical variables, and laboratory findings were compared. Therapeutic drug monitoring of steady-state plasma NVP concentrations and genotype analysis of *CYP2B6* 516 were also performed in 22 participants. The primary endpoint was continued virological suppression at the end of the study. Secondary endpoints were time to loss of virological response and adverse events.

**Results:**

During a mean follow-up of 18.4 months, the NVP-XR group demonstrated similar success at maintaining virological response compared with the NVP-IR group (82.9% vs. 85.7%; *P* = 0.72). Cox regression analysis indicated that there were no significant differences between NVP regimens for time to loss of virological response (hazard ratio: 0.940; *P* = 0.754). Furthermore, there were no significant differences in adverse events between these two groups. In the 22 participants, there was a non-significantly lower level of steady-state plasma NVP concentrations in the NVP-XR group than in NVP-IR recipients (5145.0 ng/mL vs. 6775.0 ng/mL; *P* = 0.267). The prevalence of *CYP2B6* 516 GT was 86.6%, and there was no significant difference in the distribution of *CYP2B6* 516 between these two groups.

**Conclusions:**

We found that switching from NVP-IR to NVP-XR appeared to have similar safety and efficacy compared with continuing NVP-IR among virologically suppressed, HIV-infected Taiwanese patients. Our finding of higher C_trough_ levels in both groups compared with other studies conducted in Caucasian populations and the high prevalence of *CYP2B6* 516 GT requires further investigation in a larger Taiwanese cohort.

**Electronic supplementary material:**

The online version of this article (doi:10.1186/s12879-017-2371-3) contains supplementary material, which is available to authorized users.

## Background

Nevirapine immediate-release (NVP-IR) has been used with a high level of efficacy and safety since its introduction in 1996 in the United States and 1998 in Europe as a potent non-nucleoside reverse transcriptase inhibitor (nNRTI) used in combined antiretroviral therapy (cART) [1–3]. However, efforts to simplify the regimens in terms of pill counts and frequency have continued to improve adherence to HAART [4, 5]. Therefore, although the licensed dosage for NVP-IR is 200 mg twice daily after a 200 mg daily lead-in period, an off-label maintenance dose of 400 mg (2 × 200 mg) NVP-IR has been adopted to improve compliance [6]. Clinical trials comparing a single daily dose of 400 mg NVP-IR versus 200 mg twice daily demonstrated non-inferior efficacy for the new treatment [6–8]. To further simplify treatment regimens, a new extended-release (XR) formulation was developed [9, 10]. In phase III clinical trials, 400 mg NVP-XR once daily was found not inferior to 200 mg NVP-IR twice daily in terms of virological suppression in treatment-naïve patients (VERxVE study) [11] and in virologically suppressed patients who were switched from NVP-IR to NVP-XR (TRANxITION study) [12].

Nevirapine is pharmacologically characterized by a strong relationship between NVP trough plasma concentrations and virological response, [13, 14] and a target therapeutic trough concentration of 3.0 mg/L has been proposed as a minimum effective concentration [15] because the risk of virological failure increases 5-fold (relative risk 5.0, 95% CI 1.8–13.7) with NVP C_trough_ ≤ 3 mg/mL compared with patients who had higher trough concentrations [13, 14].

However, in routine clinical practice, optimal plasma concentrations of NVP are reached in only 27.8% patients [16]. The significant inter-individual variability in plasma concentrations of nNRTI could be partially explained by differences in ethnicity, polymorphisms in enzymes, drug–drug interactions, and body weight [17–19]. Polymorphisms of *CYP2B6* may influence the metabolism of NVP, with the variant *CYP2B6* 516 GT polymorphism resulting in reduced clearance of NVP compared with *CYP2B6* 516 GG [20]. In a 2NN pharmacokinetic (PK) substudy, NVP clearance was reduced by 19.4% in patients from Thailand and South Africa compared with Caucasian and Hispanic patients [20]. This difference has been related to *CYP2B6* 516 GT polymorphisms, resulting in a 15.3% reduced clearance of NVP compared with patients with *CYP2B6* 516 GG [20]. However, a recent study of 171 HIV-infected patients in northern Taiwan showed a predominance of homozygous 516 GG alleles (66.1%), [21] which may lead to reduced NVP C_trough_ compared with patients with *CYP2B6* 516 GT. Furthermore, a recent prospective study of 227 HIV-infected, treatment-naïve patients conducted in China recommends a higher target therapeutic C_trough_ (3.9 μg/mL) of NVP for Chinese patients than the currently recommended level (3.0 μg/mL), which is predominately from the results of studies among Caucasian and African American patients [22].

In addition, previous PK studies of NVP have demonstrated a lower C_trough_ with 400 mg NVP-XR once daily compared with 200 mg NVP-IR twice daily [11, 12, 23]. Therefore, the question remains whether the non-inferior efficacy and safety results of switching virologically suppressed HIV-1-infected patients from NVP-IR to NVP-XR demonstrated in the TRANxITION study conducted in Europe and North America [12] are also applicable to virologically suppressed HIV-infected patients in Taiwan.

The current study, conducted at a large teaching medical center in southern Taiwan, was designed to evaluate the efficacy and safety of switching virologically suppressed Taiwanese patients from 200 mg NVP-IR twice daily to 400 mg NVP-XR once daily. Therapeutic drug monitoring of plasma NVP concentrations and genotype analysis of *CYP2B6* 516 were also performed in some participants.

## Methods

### Study design and patients

This study is composed of two parts: a retrospective analysis of the efficacy and safety of switching virologically suppressed patients from 200 mg NVP-IR twice daily to 400 mg NVP-XR once daily, and a prospective analysis of the impact of CYP450 polymorphisms on plasma concentrations of nNRTI. In the first part of the study, we retrospectively examined data collected from April 1, 2013, to March 31, 2015, at Kaohsiung Veterans General Hospital (KVGH), a 1200-bed, general and tertiary care hospital located in southern Taiwan. Prior to April 2013, only NVP-IR was available at KVGH. Once NVP-XR became available at KVGH in April 2013, all patients receiving NVP-IR were queried by their physician regarding their willingness to switch to NVP-XR. Because HIV-infected patients regularly visit our infectious diseases department every 1–3 months, we retrospectively screened all HIV-infected patients who were receiving NVP-IR-containing cART during the period from April 1, 2013 to June 30, 2013. Inclusion criteria for virological suppression were patients who were receiving NVP-IR plus two nucleos(t)ide reverse-transcriptase inhibitors, for a preceding minimum of 18 weeks, with undetectable (<50 HIV-1 RNA copies/mL) HIV-1 viral load (VL) in the previous 1–4 months [12]. Exclusion criteria were age < 18 years, pregnancy, or patients whose regimen included 400 mg NVP-IR once daily/switching from NVP-IR to NVP-XR/change of the NRTI backbone of combination regimens between July 1, 2013, and March 31, 2015. Enrolled patients were further subdivided into those continuing NVP-IR twice daily and those switching to NVP-XR once daily.

For the second part of the study, we evaluated the impact of CYP450 polymorphisms on plasma concentrations of nNRTI by high-performance liquid chromatography (HPLC) in HIV-infected patients, beginning in January 1, 2014. Inclusion criteria were patients who were receiving the same nNRTI-containing cART for at least 14 days. We excluded patients who were also receiving medications other than antiretroviral agents, those who were aged <20 years, and those who did not adhere to cART. For patients who were eligible and agreed to participate, a steady-state plasma sample was obtained before administration of the next dose of nNRTI. Finally, we matched those patients enrolled in the prospective part of our study to those in the retrospective analysis.

The two parts of this study were approved by the KVGH ethics committee (VGHKS14-CT7–22 and VGHKS14-CT2–13) and adhered to the principles of the Declaration of Helsinki. Informed consent was obtained from participants who underwent therapeutic drug monitoring of plasma nNRTI concentrations and genotype analysis of CYP450 polymorphisms.

### Data collection

For each enrolled patient, the following information was extracted for analysis from computerized hospital records and chart reviews: demographic characteristics, risk factors for HIV infection, comorbidities, and cART regimen. Laboratory testing included hemograms, biochemical profiles, plasma viral load, and CD4+ T cell counts at baseline and at each follow-up visit. Viral load was determined using the Roche COBAS Amplicor assay prior to 2008 and the Roche COBAS TaqMan assay subsequently. Adverse events (AE) were also recorded. Liver function abnormalities were graded according to definitions of the Division of Acquired Immunodeficiency Syndrome (DAIDS).

### Definitions

Virological suppression was defined as HIV RNA ≤50 copies/mL. A virological blip was defined as an isolated detectable HIV RNA signal after a period of suppression, followed by a return to virological suppression [24]. Virological failure was defined as two consecutive HIV-1 RNA measurements >50 copies/mL at least 2 weeks apart (also called a “double blip”) [12]. Baseline data were defined as the most recent available test result within 3 months prior to entering the study. The date of entering the study was defined as April 1, 2013 for the NVP-IR group and as the date of switching for the NVP-XR group. The duration of patient follow-up was defined as the time from entering the study to treatment failure or to the last VL measurement within the observation period. The duration of continued virological suppression before entering the study was from the first VL measurement of ≤50 copies/mL to the date of entering the study.

### Study end points

The primary end point was the proportion of patients with continued virological response with VL <50 HIV-1 RNA copies/mL at the end of the study. Patients were classified as treatment failures at the first occurrence of virological failure or a change in NVP regimen because of AEs or other reasons, death, or loss to follow-up (LTFU), [12] which was defined as no follow-up visits within 3 months prior to the end of the study. Secondary end points were time to loss of virological response (TLOVR) and AEs.

### Therapeutic drug monitoring of steady-state plasma NVP concentrations

A steady-state plasma sample was obtained from all participants before administration of the next dose of NVP. Blood samples were centrifuged immediately after collection, and plasma was removed and stored at −20 °C until analysis. We used an HPLC technique with UV detection for analysis of NVP concentrations, as previously described [25]. For each calibration curve, six standards with concentrations ranging from 0.1 to 25 μg/mL were used. The lower limit of quantification of the assay was 0.1 μg/mL. Intra- and interassay variabilities were <5%. Data were recorded and analyzed using HPLC 2D Chem Station software (Agilent Technologies, Santa Clara, CA, USA).

### Genotype analysis of *CYP2B6* 516

Genomic DNA was isolated from peripheral blood using the PureLink Genomic DNA Mini Kit (Invitrogen, Carlsbad, CA, USA). Exon 4 of the *CYP2B6* gene was amplified by polymerase chain reaction (PCR). The primers 5′ CTTGACCTGCTGCTTCTTCC 3′ and 5′ TCCCTCTCCGTCTCCCTG 3′ were used to amplify a 204-bp fragment. PCR products were digested with *Bsr*I restriction enzyme (New England Biolabs, Inc., Beverley, MA, USA). Digestion products were then loaded on a 2% agarose gel and separated by gel electrophoresis [26, 27].

### Statistical analyses

All statistical analyses were performed using IBM SPSS software, version 22.0 (IBM Corp., Armonk, NY, USA). Categorical variables were compared between the two groups using the χ^2^ or Fisher’s exact tests, and continuous variables were compared using the independent *t*-test or Mann–Whitney U test. All tests were two-tailed, and *P* < 0.05 was considered significant. The TLOVR of these two groups was analyzed using a Kaplan–Meier survival curve, and the influence of the NVP regimen (NVP-IR twice daily vs. NVP-XR once daily) on the TLOVR was investigated using Cox regression that was adjusted for age, sex, history of AIDS, cART regimen, and duration of virological suppression.

## Results

### Demographic characteristics and comorbidities of study participants

**Fig. 1:**
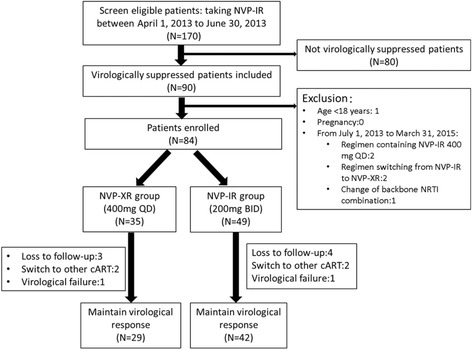
Study design

We retrospectively screened the records of 170 patients who were receiving NVP-IR during the period April 1 to June 30, 2013. Ninety met the inclusion criteria for virological suppression. Overall, six of these patients were excluded because they were <18 years old (*n* = 1), receiving a regimen that contained 400 mg NVP-IR once daily (*n* = 2), switching from NVP-IR to NVP-XR (*n* = 2), or had a change of the NRTI backbone (*n* = 1) between July 1, 2013, and March 31, 2015 (Fig. 1). Of the 84 patients enrolled in this study, 49 (59%) continued a regimen of NVP-IR twice daily and 35 (41%) were switched to an NVP-XR once daily regimen during the period from April 1 to June 30, 2013.

The baseline demographic characteristics and comorbidities of the two participant groups are shown in Table 1. The median age was 41 years (SD ± 13 years); 72 participants (85.7%) were male. There were no statistically significant differences between the NVP-IR and NVP-XR groups with respect to age, sex, risk factors for HIV, comorbidities, duration of continued virological suppression, history of a virologic blip, baseline CD4+ counts, or patient follow-up. However, the two groups differed significantly in the NRTI backbone of combination regimens at baseline. Most patients in the NVP-IR group were treated with lamivudine and zidovudine (Combivir®) (NVP-IR vs. NVP-XR: 55.1% vs. 0%; *P* < 0.001) whereas most patients in the NVP-XR group received abacavir and lamivudine (Kivexa®) (NVP-XR vs. NVP-IR: 77.1% vs. 32.7%; *P* < 0.001). Regarding the third drug of cART before entering the study, 24 of 35 patients (68.6%) in the NVP-XR group received an off-label regimen with 400 mg NVP-IR once daily, and all 49 patients in the NVP-IR group received 200 mg NVP-IR twice daily.

**Table 1:** Demographic characteristics and co-morbidities for the 84 virologically suppressed, HIV-infected patients entered in this study

	All	NVP-XR	NVP-IR	*P*
	*N* = 84	*N* = 35	*N* = 49	
Age, mean years (± SD)	41 (13)	43 (12)	40 (14)	0.38
Gender, Male (%)	72 (85.7)	30 (85.7)	42 (85.7)	1.0
Body weight, kg (± SD)	66.3 (12.3)	67.1 (13.1)	65.7 (11.8)	0.62
Risk factor				
MSM, n (%)	64 (82.1)	29 (82.8)	35 (71.4)	0.22
Heterosexual, n (%)	10 (11.9)	3 (8.6)	7 (14.3)	0.51
IVDU, n (%)	9 (10.7)	3 (8.6)	6 (12.2)	0.73
Vertical transmission, n (%)	1 (1.1)	0 (0)	1 (2.0)	1.0
Underlying disease				
Diabetes mellitus, n (%)	3 (3.6)	0 (0.0)	3 (6.1)	0.26
CKD, n (%)	0 (0.0)	0 (0.0)	0 (0.0)	
Autoimmune disease, n (%)	0 (0.0)	0 (0.0)	0 (0.0)	
Chronic hepatitis B infection, n (%)	8 (9.5)	4 (11.4)	4 (8.2)	0.71
Chronic hepatitis C infection, n (%)	6 (7.1)	1 (2.9)	5 (10.2)	0.39
Backbone of cART				<0.001
Kivexa, n (%)	43 (51.2)	27 (77.1)	16 (32.7)	<0.001
Combivir, n (%)	27 (32.1)	0 (0.0)	27 (55.1)	<0.001
TDF+ 3TC, n (%)	8 (9.5)	4 (11.4)	4 (8.1)	0.71
DDI + 3TC, n (%)	5 (6.0)	4 (11.4)	1 (2.0)	0.16
Kaletra, n (%)	1 (1.2)	0 (0.0)	1 (2.0)	1.0
Third drug of cART^a^				
NVP-IR 200 mg twice daily, n (%)	60 (71.4)	11 (31.4)	49 (100)	<0.001
NVP-IR 400 mg once daily, n (%)	24 (28.6%)	24 (68.6)	0 (0.0)	<0.001
Duration of continued virological suppression at entering this study, mean days (±SD)	993 (758)	1042 (565)	959 (875)	0.60
History of virological blip before entering this study, n (%)	10 (11.9)	3 (8.6)	7 (14.3)	0.51
Baseline CD4, mean cells/mm^3^ (±SD)	489 (244)	463 (193)	508 (276)	0.41
Patient follow-up, days (±SD)	552 (170)	555 (172)	549 (170)	0.86

*MSM* men who have sex with men, *CKD* chronic kidney disease, *DDI* didanosine, *IVDU* intravenous drug user, *NVP* nevirapine, *SD* standard deviation, *TDF* tenofovir, 3*TC* lamivudine

^a^In the NVP-XR group, this means the nNRTIs prescribed prior to switching to NVP-XR-containing HAART

### Outcomes of primary and secondary study end points

Compared with those in the NVP-IR group, virologically suppressed HIV-infected participants in the NVP-XR group demonstrated more or less similar success at maintaining virological response during the mean follow-up time of 18.4 months (NVP-XR vs. NVP-IR: 82.9% vs. 85.7%; *P* = 0.72) (Table 2). No patients in either group died during the observation period. In the NVP-XR group, one patient developed virological failure, which may have been as a result of poor compliance with cART, according to the chart review. Two patients were withdrawn from NVP-XR: one was suspected of having a hypersensitivity reaction to NVP-XR, and the other was owing to an insufficient supply of the drug. Three patients in the NVP-XR group were lost to follow-up for unknown reasons. In the NVP-IR group, one patient suffered from virological failure. Two patients were withdrawn from NVP-IR because they had NVP-induced hepatitis (one with DAIDS grade 2 and the other, grade 3). Four patients in the NVP-IR group were lost to follow-up. During the observation period, six patients (7.1%) experienced isolated viral blips after entering the study (NVP-XR vs. NVP-IR: 11.4% vs. 4.1%; *P* = 0.23). There were no differences in CD4+ count changes between the two groups (NVP-XR vs. NVP-IR: 23 cells/mm^3^ vs. 76 cells/mm^3^; *P* = 0.32).

**Table 2:** Outcomes for the 84 virologically suppressed HIV-infected patients entered in this study

	All	NVP-XR	NVP-IR	*P*
	*N* = 84	*N* = 35	*N* = 49	
Ability to maintain a virological response, n (%)	71 (84.5)	29 (82.9)	42 (85.7)	0.72
Reason for treatment failure				
Death, n (%)	0 (0.0)	0 (0.0)	0 (0.0)	
Switch to other cART due to adverse effect or other cause, n (%)	4 (4.8)	2 (5.7) ^a^	2 (4.1)	1.00
Virological failure, n (%)	2 (2.4)	1 (2.9)	1 (2.0)	1.00
Loss to follow-up, n (%)	6 (7.1)	3 (8.6)	4 (8.1)	1.00
Virological blip, n (%)	6 (7.1)	4 (11.4)	2 (4.1)	0.23
Change from baseline in CD4 T-cells, mean cells/mm^3^ (± SD)	14 (122)	23 (171)	76 (127)	0.33

*cART* combined antiretroviral therapy, *NVP* nevirapine, *SD* standard deviation

^a^One patient in the NVP-XR group discontinued NVP-XR because of insufficient supply of the drug

**Fig. 2:**
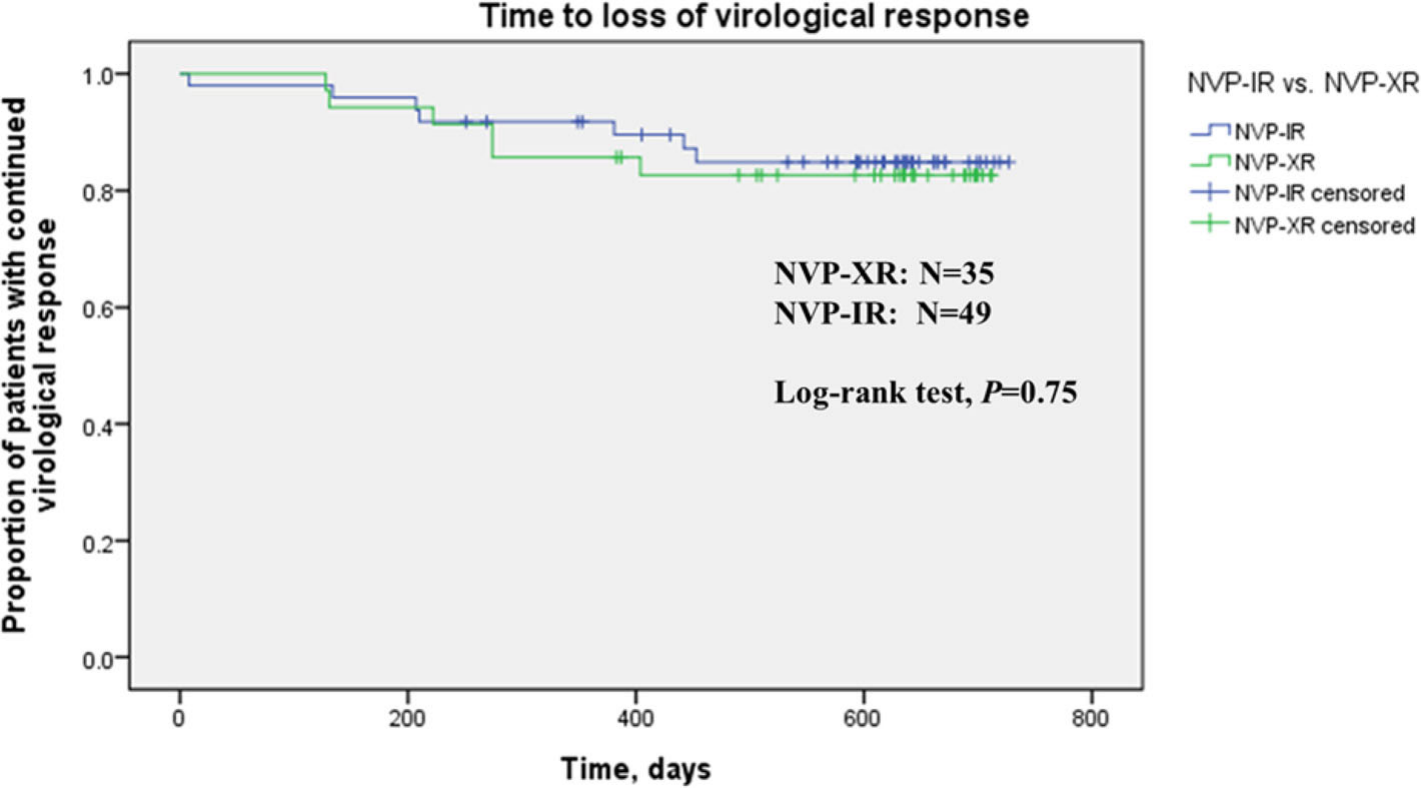
Kaplan–Meier survival curves, time to loss of virological response in NVP-XR and NVP-IR groups. No significant difference was detected between the two treatment groups by log-rank test (*P* = 0.75)

The TLOVR for the two groups is shown in Fig. 2. There were no differences between these groups by log-rank test (*P* = 0.75). A Cox model adjusted for age, sex, history of AIDS, cART regimen, and duration of virological suppression demonstrated a non-significant difference between the NVP regimens with respect to hazard ratios (HR) for TLOVR (NVP-XR vs. NVP-IR: HR 0.940, 95% CI 0.254–3.484; *P* = 0.926) (Additional file 1).

### Adverse events among study participants

There were no significant differences in AEs between the NVP-XR and NVP-IR groups (Table 3). Observed AEs included gastrointestinal complaints, skin rashes, and liver function abnormalities. One patient in the NVP-XR group found remnants of NVP-XR tablets in his stool. Three of 84 patients (3.6%) discontinued their NVP regimen owing to AEs (Table 2). One patient taking NVP-XR experienced impaired liver function (DAIDS grade 1), low-grade fever, and skin rash 2 weeks after switching, suggesting a hypersensitivity reaction; two patients taking NVP-IR experienced NVP-induced hepatitis (DAIDS grade 2 and grade 3) that improved after switching to atazanavir. The majority of liver function abnormalities were mild (DAIDS grade 1). Higher grade liver function abnormalities (DAIDS grade ≥ 2) were infrequent (NVP-XR vs. NVP-IR: 2.9% vs. 4.1%; *P =* 1.0). There was one case (2.9%) of grade 4 aspartate transaminase/alanine transaminase levels in the NVP-XR group.

**Table 3:** Adverse effects and liver function abnormality tests of the 84 virologically suppressed HIV-infected patients entered in this study

a Comparison of adverse effects between NVP-XR and NVP-IR									
	All *N* = 84	NVP-XR *N* = 35			NVP-IR *N* = 49				*P*
Skin rash, n (%)	1 (1.2)	1 (2.9)			0 (0.0)				0.42
Gastrointestinal disorders, n (%)	2 (2.4)	1 (2.9)			1 (2.0)				1.0
Tablet remnants in stools, n (%)	1 (2.4)	1 (2.9)			0 (0.0)				0.42
b Comparison of liver function abnormalities between NVP-XR and NVP-IR									
	NVP-XR 400 mg qd (*N* = 35)				NVP-IR 200 mg bid (*N* = 49)				*P*
AST elevation, n (%)	6 (17.1)				12 (24.5)				0.42
	Grade I	Grade II	Grade III	Grade IV	Grade I	Grade II	Grade III	Grade IV	
	n (%)	n (%)	n (%)	n (%)	n (%)	n (%)	n (%)	n (%)	
	5 (14.3)	0 (0)	0 (0)	1 (2.9)	10 (22.4)	1 (2.0)	1 (2.0)	0 (0)	0.28
ALT elevation, n (%)	9 (25.7)				16 (32.7)				0.49
	Grade I	Grade II	Grade III	Grade IV	Grade I	Grade II	Grade III	Grade IV	
	n (%)	n (%)	n (%)	n (%)	n (%)	n (%)	n (%)	n (%)	
	8 (22.9)	0 (0)	0 (0)	1 (2.9)	13 (28.6)	2 (4.1)	1 (2.0)	0 (0)	
Bilirubin, n (%)	1 (2.9)				0 (0)				0.42
	Grade I	Grade II	Grade III	Grade IV	Grade I	Grade II	Grade III	Grade IV	
	n (%)	n (%)	n (%)	n (%)	n (%)	n (%)	n (%)	n (%)	
	1 (2.9)	0 (0)	0 (0)	0 (0)	0 (0)	0 (0)	0 (0)	0 (0)	

*AST* aspartate aminotransferase, *ALT* alanine aminotransferase

(a) Comparison of adverse effects between NVP-XR (*n* = 35) and NVP-IR (*n* = 49). (b) Comparison of liver function abnormalities between NVP-XR (*n* = 35) and NVP-IR (*n* = 49)

### Therapeutic drug monitoring of steady-state plasma NVP concentration: Comparison between NVP-XR and NVP-IR groups

A total of 48 patients at KVGH were enrolled, to evaluate the impact of *CYP2B6* 516 polymorphisms on plasma concentrations of NVP from January 1, 2014 to December 31, 2014. A total of 22 patients (22/48, 45.83%, Additional file 2) were matched to patients enrolled in the retrospective part of the current study (22/84, 26.19%), eight patients from the NVP-IR group and 14 from the NVP-XR group. There were no significant differences between these groups in age, sex, height, or weight (Additional file 3). Steady-state plasma NVP concentrations were lower in the NVP-XR group, but this difference did not reach statistical significance (NVP concentration (interquartile range): NVP-XR vs. NVP-IR, 5145.0 ng/mL (4070.0–5740.0 ng/mL) vs. 6775.0 ng/mL (4925.0–8380.0 ng/mL); *P* = 0.267) (Additional file 3).

### Genotype analysis of *CYP2B6* 516: Comparison between NVP-XR and NVP-IR groups

Among the 22 patients participating in monitoring of steady-state plasma NVP concentrations, genotype analysis of *CYP2B6* 516 was conducted in 15 (15/22, 68.2%) (Additional file 4); genotype analysis was not performed in the remaining seven patients owing to samples with insufficient residual volume. The prevalence of *CYP2B6* 516 GT was 86.6% (13/15). There was no significant difference in the distribution of *CYP2B6* 516 between these two groups (Additional file 3).

## Discussion

To the best of our knowledge, this small retrospective cohort is the first to investigate the safety and efficacy of switching to NVP-XR from NVP-IR among virologically suppressed HIV-infected patients in Taiwan. Although the TRANxITION study demonstrated non-inferior efficacy and safety of switching virologically suppressed HIV-1-infected patients from NVP-IR to NVP-XR, the results were derived mainly from a Caucasian population. In this retrospective cohort study of virologically suppressed HIV-infected Taiwanese patients, we found that the NVP-XR group was similar to the NVP-IR group with respect to maintaining a virological response. The difference may not have been significant because the numbers of patients were relatively small and the study lacked the power to demonstrate a statistically significant difference. Despite methodological differences, the results of our comparative analyses of virological response indicated worse results compared with those of the TRANxITION study [12]. During NVP-IR therapy, 85.7% and 92.6% of patients in the current and TRANxITION study, respectively, were virologically suppressed. During NVP-XR therapy, 82.9 and 93.6% of patients in the current and TRANxITION studies, respectively, were virologically suppressed. The lower rate of virological response in our study may result from a longer follow-up period, as the mean follow-up times for the current and TRANxITION studies were respectively 18.4 and 6 months. Furthermore, the current study was conducted outside a well-controlled trial setting. Therefore, the efficacy of NVP-IR and NVP-XR in maintaining virological response may be underestimated here owing to a relatively high proportion of LTFU treatment failures in both groups (NVP-IR vs. NVP XR: 50% (3/6) vs. 57% (4/7)), which is inherent to retrospective studies.

Because previous studies have demonstrated a significant relationship between NVP trough plasma concentrations and virological response, [13, 28] we further investigated the NVP trough concentrations in both NVP-IR and NVP-XR groups. The median of trough plasma concentrations was 6775.0 ng/mL for NVP-IR and 5145.0 ng/mL for NVP-XR in our study (Additional file 3). The plasma concentrations of NVP in both groups are higher than the C_trough_ of 3.0 mg/L recommended in most guidelines [15] or C_trough_ of 3.9 μg/mL for Chinese patients in a recent study [22]. The non-significant difference of NVP trough concentrations between these two groups may be owing to the small sample size in each group. This finding is compatible with previous studies, which demonstrated a lower C_trough_ with NVP-XR therapy compared with 200 mg NVP-IR twice daily [11, 23].

We believe that there was no obvious selection bias between patients receiving NVP-XR and NVP-IR who also underwent therapeutic drug monitoring of steady-state plasma NVP concentration in the current study. Compliance, drug–drug interactions, and differences in pharmacokinetics are critical factors that influence the plasma concentrations of antiretroviral drugs [29]. Our patient population was assumed to be reasonably compliant because they had maintained a stable virological response to NVP-containing cART prior to entering the study, and all participants remained virologically suppressed for at least 1–4 months (duration of continued virological suppression at entering this study, days (±SD): NVP-XR vs. NVP-IR, 1042 (565) vs. 959 (875); *P* = 0.60). Additionally, these patients only received antiretroviral agents and did not take any other prescribed medicine concomitantly. Finally, genotype analysis of *CYP2B6* 516 in 15 patients also revealed no significant difference in *CYP2B6* 516 polymorphisms between these two groups.

Surprisingly, however, our study demonstrated higher C_trough_ levels in both groups compared with other studies conducted in the Netherlands, [30] Germany, [31] and Canada [32]. This difference may result from a higher prevalence of *CYP2B6* 516 GT in our study population (86.6%) compared with other reports of Han Chinese, [33] Caucasians, [29] and Taiwanese in northern Taiwan [21]. Our finding of a high prevalence of *CYP2B6* 516 GT in the current study requires further verification in a larger Taiwanese population.

Maximal and durable suppression of plasma HIV viral load has now been established as the primary aim of antiretroviral treatment for both treatment-naïve and treatment-experienced patients [24]. However, viral replication is still not fully controlled in all patients at all times, and transient viremia, often between 50 and 400 copies/mL (a virological blip) has been frequently reported [34]. However, the impact of HIV RNA blips on virological failure remains controversial [35, 36]. In the ACTG 343 and Merck 035 trials, treatment-experienced patients who had virological blips did not have an increased risk of virological failure compared with patients with continuing virological suppression [35]. However, the Swiss HIV Cohort and Frankfurt HIV Clinic Cohort studies showed a slightly increased risk of virological failure for patients with one or more virological blips compared with viral suppression (HR 2.01, 95% CI 1.51–2.91; *P* < 0.0001) [36]. In our cohort study, six of 84 patients (7.1%) experienced a virologic blip during the 18.4-month follow-up. In Cox regressions adjusted for age, sex, history of AIDS, cART regimen, and history of virological suppression, the HR of viral blips was 1.023 (*P* = 1.00) for the NVP-XR group compared with the NVP-IR group. The relationship between viral blip and virological failure in Taiwanese patients requires further investigation in a larger population.

The current study has several limitations and biases inherent to retrospective cohort studies, the first being selection bias. Second, there were significant differences in cART backbone drugs between the NVP-XR and NVP-IR groups. However, the CNA30024 study comparing the durability of viral effect and the safety profile of triple therapy with either abacavir or zidovudine, combined with lamivudine and efavirenz, demonstrated similar virological suppression (HIV RNA ≤ 50 copies/mL) in treatment-naïve patients at week 48 (abacavir group vs. zidovudine group: 70% vs. 69%) [37]. Third, it is difficult to determine patient compliance with medications from retrospective chart reviews. This is important because adherence to treatment is closely related to sustained viral suppression [38]. As mentioned above, we believe that our patient population was reasonably compliant because only patients with virological suppression for at least 1–4 months were included. Finally, the frequency of adverse reactions to NVP in both groups may be underestimated. Although the frequency of moderate to severe liver function abnormalities is similar to that of the TRANxITION study, it is likely that we missed some low-grade skin rashes and gastrointestinal disorders that did not require modification of the antiretroviral regimen.

## Conclusions

In this retrospective study, we found that switching from cART regimens with twice-daily doses of 200 mg NVP-IR to a once-daily dose of 400 mg NVP-XR appeared nearly as safe and effective as continuing NVP-IR in virologically suppressed HIV-infected Taiwanese patients. Our findings of higher C_trough_ levels in both groups compared with other studies conducted among Caucasian populations may be due to the high prevalence of *CYP2B6* 516 intermediate/low metabolizers in the present study.

## Additional files

Additional file 1: Multivariate analysis of treatment failure in 84 virologically suppressed HIV-infected patients. In Cox regression, the hazard ratios for TLOVR between NVP regimen (NVP-XR vs. NVP-IR) was 0.940 (95% CI, 0.254–3.484; *P* = 0.926). (DOCX 14 kb)

Additional file 2: Comparison of demographic characteristics, steady-state plasma concentration of NVP, and genotype analysis of *CYP2B6* 516 in 48 HIV-infected patients enrolled in KVGH from January 1, 2014 to December 31, 2014, by patients enrolled or not enrolled in the retrospective part of the current study. The 22 patients enrolled in the retrospective analysis of the current study were older (37 vs. 27.5, *P* < 0.001) and heavier (74.5 kg vs. 64.8 kg, *P* = 0.021) than the 26 patients not enrolled in the retrospective analysis of the current study. There were no significant differences between these two groups in sex, height, the steady-state plasma NVP concentration, and genotype analysis of *CYP2B6* 516. (DOCX 15 kb)

Additional file 3: Demographic characteristics, steady-state plasma concentration of NVP, and genotype analysis of *CYP2B6* 516 of 22 virologically suppressed, HIV-infected patients enrolled in this study, by NVP regimens. There were no significant differences between NVP-XR and NPV-IR in age, sex, height, weight, steady-state plasma NVP concentrations, and genotype analysis of *CYP2B6* 516. (DOCX 15 kb)

Additional file 4: PCR-restriction fragment length polymorphism analysis of *CYP2B6* 516. Three cases (A, B, C) participating in genotype analysis of *CYP2B6* 516 in the current study are shown. Lane M represents a size marker. After digestion with *Bsr*I restriction enzyme, wild-type GG (case B) is visible as one band of 152 base pair. Heterozygous GT (case C) is visible as two bands (152 base pair and 204 base pair); homozygous mutant TT (case A) is visible as one band of 204 base pair. (PPTX 122 kb)

## Abbreviations

AE: Adverse events
cART: Combined antiretroviral therapy
DAIDS: Division of Acquired Immunodeficiency Syndrome
HAART: Highly active antiretroviral therapy
HPLC: High-performance liquid chromatography
HR: Hazard ratio
KVGH: Kaohsiung Veterans General Hospital
LTFU: lost to follow-up
NRTI: Nucleoside reverse transcriptase inhibitor
NVP-IR: Nevirapine immediate-release
NVP-XR: Nevirapine extended-release
PCR: Polymerase chain reaction
PK: Pharmacokinetic
TLOVR: Time to loss of virological response
VL: Viral load
