# Supplementary material for: Efficacy and safety of switching from nevirapine immediate-release twice daily to nevirapine extended-release once daily in virologically suppressed HIV-infected patients: a retrospective cohort study in Taiwan
Source: BMC Infect Dis. 2017 Apr 11;17:261. doi: 10.1186/s12879-017-2371-3 (PMC5387218; doi:10.1186/s12879-017-2371-3)
Supplement: Supplementary file 1 — Multivariate analysis of treatment failure in 84 virologically suppressed HIV-infected patients. In Cox regression, the hazard ratios for TLOVR between NVP regimen (NVP-XR vs. NVP-IR) was 0.940 (95% CI, 0.254–3.484; P = 0.926). (DOCX 14 kb) [file 12879_2017_2371_MOESM1_ESM.docx]

**Supplementary file 1. Multivariate analysis of treatment failure in 84 virologically suppressed HIV-infected patients.**

|  | Progression to treatment failure | | | |
| --- | --- | --- | --- | --- |
| Variable | HR | 95% CI | | *P* |
| Male vs. female | 2.838 | 0.745 | 10.802 | 0.126 |
| Age | 1.040 | 0.999 | 1.082 | 0.053 |
| Duration of virological suppression | 1.000 | 0.999 | 1.000 | 0.364 |
| History of AIDS | 1.356 | 0.387 | 4.760 | 0.634 |
| NVP-XR vs. NVP-IR | 0.940 | 0.254 | 3.484 | 0.926 |
| Combivir-containing cART | 0.435 | 0.076 | 2.476 | 0.348 |
| Kivexa-containing cART | 0.355 | 0.084 | 1.504 | 0.160 |

AIDS, acquired immune deficiency syndrome; cART, combined antiretroviral therapy; NVP, nevirapine.
