# Supplementary material for: Efficacy and safety of switching from nevirapine immediate-release twice daily to nevirapine extended-release once daily in virologically suppressed HIV-infected patients: a retrospective cohort study in Taiwan
Source: BMC Infect Dis. 2017 Apr 11;17:261. doi: 10.1186/s12879-017-2371-3 (PMC5387218; doi:10.1186/s12879-017-2371-3)
Supplement: Supplementary file 2 — Comparison of demographic characteristics, steady-state plasma concentration of NVP, and genotype analysis of CYP2B6 516 in 48 HIV-infected patients enrolled in KVGH from January 1, 2014 to December 31, 2014, by patients enrolled or not enrolled in the retrospective part of the current study. The 22 patients enrolled in the retrospective analysis of the current study were older (37 vs. 27.5, P < 0.001) and heavier (74.5 kg vs. 64.8 kg, P = 0.021) than the 26 patients not enrolled in the retrospective analysis of the current study. There were no significant differences between these two groups in sex, height, the steady-state plasma NVP concentration, and genotype analysis of CYP2B6 516. (DOCX 15 kb) [file 12879_2017_2371_MOESM2_ESM.docx]

**Supplementary file 2. Comparison of demographic characteristics, steady-state plasma concentration of NVP, and genotype analysis of *CYP2B6* 516 in 48 HIV-infected patients enrolled in KVGH from January 1, 2014 to December 31, 2014, by patients enrolled or not enrolled in the retrospective part of the current study.**

|  | | All  N = 48 | Enrolled  N = 22 | Not enrolled  N = 26 | *P* |
| --- | --- | --- | --- | --- | --- |
| Median age, years (IQR) | | 33 (27-37) | 37 (34-45) | 27.5 (24-33) | <0.001 |
| Male, n (%) | | 46 (95.8) | 21 (95.5) | 25 (96.2) | 1.000 |
| Median height, cm (IQR) | | 172.0 (170.0-176.0) | 171.5 (170.0-176.0) | 173.0 (170.0-176.0) | 0.897 |
| Median body weight, kg (IQR) | | 68.0 (59.0-77.2) | 74.5 (63.0-84.0) | 64.8 (58.0-73.0) | 0.021 |
| Median NVP serum concentration, ng/mL (IQR) | | 6100.0 (4935.0-7470.0) | 5215.0 (4850.0-7120.0) | 6615.0 (5060.0-7480.0) | 0.277 |
| Genotype analysis of *CYP2B6* 516^*^ | | 35 | 15 | 20 | 0.509 |
|  | GG, n (%) | 4 (11.4) | 1 (6.7) | 3 (15) |  |
|  | GT, n (%) | 27 (77.1) | 13 (86.6) | 14 (70) |  |
|  | TT, n (%) | 4 (11.4) | 1 (6.7) | 3 (15) |  |

^*^Among the 48 patients participating in monitoring steady-state plasma concentration of NVP, genotype analysis of *CYP2B6* 516 was conducted in 35 patients (enrolled group vs. not-enrolled group: 15 vs. 20).
