# Supplementary material for: Efficacy and safety of switching from nevirapine immediate-release twice daily to nevirapine extended-release once daily in virologically suppressed HIV-infected patients: a retrospective cohort study in Taiwan
Source: BMC Infect Dis. 2017 Apr 11;17:261. doi: 10.1186/s12879-017-2371-3 (PMC5387218; doi:10.1186/s12879-017-2371-3)
Supplement: Supplementary file 3 — Demographic characteristics, steady-state plasma concentration of NVP, and genotype analysis of CYP2B6 516 of 22 virologically suppressed, HIV-infected patients enrolled in this study, by NVP regimens. There were no significant differences between NVP-XR and NPV-IR in age, sex, height, weight, steady-state plasma NVP concentrations, and genotype analysis of CYP2B6 516. (DOCX 15 kb) [file 12879_2017_2371_MOESM3_ESM.docx]

**Supplementary file 3. Demographic characteristics, steady-state plasma concentration of NVP, and genotype analysis of *CYP2B6* 516 of 22 virologically suppressed, HIV-infected patients enrolled in this study, by NVP regimens.**

|  | | All  N = 22 | NVP-XR  N = 14 | NVP-IR  N = 8 | *P* |
| --- | --- | --- | --- | --- | --- |
| Median age, years (IQR) | | 37 (34-45) | 37 (35-45) | 39 (31.5-41.5) | 0.616 |
| Male, n (%) | | 21 (95.5) | 13 (92.9) | 8 (100) | 0.616 |
| Median height, cm (IQR) | | 171.5 (170.0-176.0) | 171.5 (170.0-176.0) | 172.5 (169.0-177.0) | 0.815 |
| Median body weight, kg (IQR) | | 74.5 (63.0-84.0) | 73.6 (63.0-86.0) | 74.5 (66.0-80.5) | 0.815 |
| Median NVP serum concentration, ng/mL (IQR) | | 5215.0 (4850.0-7120.0) | 5145.0 (4070.0-5740.0) | 6775.0 (4925.0-8380.0) | 0.267 |
| Genotype analysis of *CYP2B6* 516^*^ | | 15 | 10 | 5 | 0.099 |
|  | GG, n (%) | 1 (6.7) | 0 (0) | 1 (20) |  |
|  | GT, n (%) | 13 (86.6) | 10 (100) | 3 (60) |  |
|  | TT, n (%) | 1 (6.7) | 0 (0) | 1 (20) |  |

^*^Among the 22 patients participating in monitoring steady-state plasma concentration of NVP, genotype analysis of *CYP2B6* 516 was conducted in 15 (NVP-XR vs. NVP-IR: 10 vs. 5).
