# Supplementary figures and images for: Efficacy and safety of switching from nevirapine immediate-release twice daily to nevirapine extended-release once daily in virologically suppressed HIV-infected patients: a retrospective cohort study in Taiwan
Source: BMC Infect Dis. 2017 Apr 11;17:261. doi: 10.1186/s12879-017-2371-3 (PMC5387218; doi:10.1186/s12879-017-2371-3)

## Slide 1
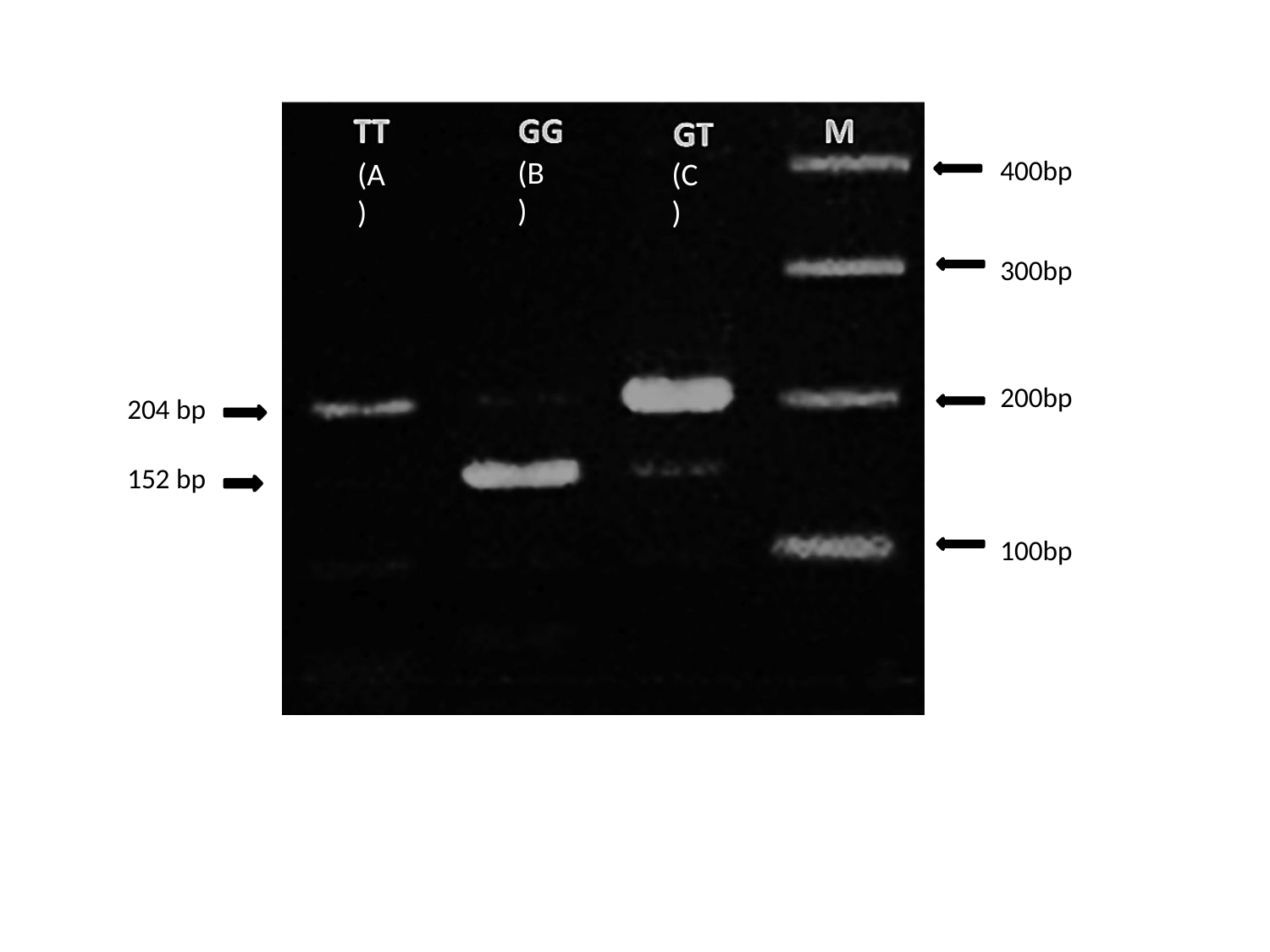

(B)
400bp
(C)
(A)
300bp
200bp
204 bp
152 bp
100bp

Supplement: Supplementary file 4 — PCR-restriction fragment length polymorphism analysis of CYP2B6 516. Three cases (A, B, C) participating in genotype analysis of CYP2B6 516 in the current study are shown. Lane M represents a size marker. After digestion with BsrI restriction enzyme, wild-type GG (case B) is visible as one band of 152 base pair. Heterozygous GT (case C) is visible as two bands (152 base pair and 204 base pair); homozygous mutant TT (case A) is visible as one band of 204 base pair. (PPTX 122 kb) [file 12879_2017_2371_MOESM4_ESM.pptx]
